# Prediction and interpretation of miRNA-disease associations based on miRNA target genes using canonical correlation analysis

**DOI:** 10.1186/s12859-019-2998-8

**Published:** 2019-07-25

**Authors:** Hailin Chen, Zuping Zhang, Dayi Feng

**Affiliations:** 1 School of Software, East China Jiaotong University, Nanchang, 330013 China; 2 School of Computer Science and Engineering, Central South University, Changsha, 410083 China

**Keywords:** miRNA-disease associations, Target genes, Canonical correlation analysis

## Abstract

**Background:**

It has been shown that the deregulation of miRNAs is associated with the development and progression of many human diseases. To reduce time and cost of biological experiments, a number of algorithms have been proposed for predicting miRNA-disease associations. However, the existing methods rarely investigated the cause-and-effect mechanism behind these associations, which hindered further biomedical follow-ups.

**Results:**

In this study, we presented a CCA-based model in which the possible molecular causes of miRNA-disease associations were comprehensively revealed by extracting correlated sets of genes and diseases based on the co-occurrence of miRNAs in target gene profiles and disease profiles. Our method directly suggested the underlying genes involved, which could be used for experimental tests and confirmation. The inference of associated diseases of a new miRNA was made by taking into account the weight vectors of the extracted sets.

We extracted 60 pairs of correlated sets from 404 miRNAs with two profiles for 2796 target genes and 362 diseases. The extracted diseases could be considered as possible outcomes of miRNAs regulating the target genes which appeared in the same set, some of which were supported by independent source of information. Furthermore, we tested our method on the 404 miRNAs under the condition of 5-fold cross validations and received an AUC value of 0.84606. Finally, we extensively inferred miRNA-disease associations for 100 new miRNAs and some interesting prediction results were validated by established databases.

**Conclusions:**

The encouraging results demonstrated that our method could provide a biologically relevant prediction and interpretation of associations between miRNAs and diseases, which were of great usefulness when guiding biological experiments for scientific research.

**Electronic supplementary material:**

The online version of this article (10.1186/s12859-019-2998-8) contains supplementary material, which is available to authorized users.

## Background

microRNAs (miRNAs) are one category of small non-coding RNAs that regulate gene expressions by base pairing with 3^′^-UTRs of messenger RNAs (mRNAs). Since the initial discovery in 1993 [1], the number of currently annotated miRNAs has increased steadily. As of November 2018, the newest version of miRBase [2] contained > 480,000 mature miRNA sequences in 271 species. There are growing studies suggesting that miRNAs play important roles in some essential biological processes, such as cell proliferation [3], development [4], differentiation [5, 6] and metabolism [7]. Hence the dysfunction of miRNAs will result in aberrant cell behaviors, and they have been associated with the development and progression of many human diseases. For example, Nagaraja et al. [8] revealed that the overexpression of *mir-100* inhibited mTOR signaling in clear cell ovarian cancer. In addition, the conserved sequences, specific secondary structures and the ability to control gene expression makes miRNAs suitable targets for drug development [9] and recent studies [10–13] have demonstrated their application in the therapeutic exploitation.

Because of the wide-spread clinical implications, some online databases [14–16] have been established for containing experimentally confirmed evidence for associations between miRNAs and diseases via text mining. These repositories serve as comprehensive resources for studying the impacts of miRNAs on human diseases. However, our current knowledge of the involvement of miRNAs in diseases is far from completeness and thus those undiscovered associations cannot be mined from the literature. Meanwhile, experimental identification of miRNA-induced diseases by biological technology is costly and laborious. Therefore, computational prediction of the most promising miRNA-disease associations for further confirmation is receiving enormous attention.

The computational efforts made in this field can mainly be divided into two groups. The methods in the first group extensively exploited the biological evidence that miRNAs exert their functions by regulating the expression levels of their target mRNAs [17]. They first comprehensively collected two sets of genes, namely miRNA target genes and disease-related genes. The miRNA-disease associations were then inferred according to the similarity values or the interactions between the two sets of genes. For example, Mørk et al. [18] proposed a model miRPD to explictitly infer miRNA-protein-disease associations by coupling miRNA-gene associations with gene-disease associations. Li et al. [19] presented a computational framework to prioritize human cancer miRNAs by calculating the functional consistency scores (FCS) between the miRNA target genes and the cancer-related genes. Shi et al. [20] developed a computational method to identify potential miRNA-disease associations by mapping disease genes and miRNA target genes onto PPI (protein-protein interaction) networks for enrichment score calculation. These methods widely used predicted miRNA target genes to support miRNA-disease association inference. Due to high false positive rate of the predicted target genes of miRNAs, it is difficult for the above methods to achieve stable prediction results.

The methods in the other group were based on the conclusion that functionally similar miRNAs are usually involved in phenotypically similar diseases [21]. For instance, Chen et al. [22] developed a method RWRMDA to infer potential miRNA-disease associations by implementing random walks on miRNA-miRNA functional similarity network. Similarly, Chen et al. [23] devised a method to infer OMIM disease candidates related to a specific miRNA via random walks on disease similarity network. Afterwards, several methods [24–38] have been presented to infer novel miRNA-disease associations by incorporating both the miRNA similarity network and the disease similarity network. Experimental results demonstrated that combining the two types of similarity networks could improve prediction performance. However, miRNA functional similarity calculation is a major challenge needed to be properly addressed in these methods. The functional similarity between two miRNAs was usually measured based on their associated diseases, which could produce overestimated validation accuracy [37]. Moreover, these similarity-based methods do not explicitly facilitate forming hypotheses about the possible molecular causes of the miRNA-disease associations [18].

In this paper, we presented a novel method to predict potentially related diseases of miRNA candidates based on their target genes on a large scale, without limiting ourselves to similarity measurement. Experimentally supported miRNA-gene interactions and miRNA-disease associations were first obtained from existing databases to build target gene profiles and disease profiles for miRNAs. We then applied canonical correlation analysis (CCA) to extract correlated sets of genes and diseases based on the co-occurrence of miRNAs in the two profiles. For a new miRNA, its disease profiles were inferred based on the weight vectors of the extracted correlated sets. Results demonstrated that the extracted sets of genes and diseases provided meaningful explanation to the molecular causes of the miRNA-disease associations, and that diseases in each correlated set were outcomes from miRNA perturbations of target genes. When applied to collected data sets for 5-fold cross-validation experiments, our method obtained an AUC value of 0.84606. We finally conducted comprehensive miRNA-disease association predictions and confirmed some high-ranking results using independent source of information.

## Results

### Extraction of canonical component sets of miRNA-targeted genes and miRNA-related diseases

The proposed method (see Methods) was applied to the target gene profiles and disease profiles to receive 60 canonical components (CC). The extracted genes and diseases in each component were available at Additional file 1. We also provided information of miRNAs that contributed to the correlations. It could be discovered that each component includes a small number of genes and diseases, which indicated an advantage of adding parameters *c*_*1*_ and *c*_*2*_ to impose sparseness on ordinary canonical correlation analysis (OCCA). It should be noted that experiments suggested that all the weight vectors received by OCCA were not sparse with the parameter *K* ranged from 10 to 200 by 10 increments, which prevented the extraction of CCs.

### Interpretation of miRNA-disease associations through the extracted component sets

It has been known that miRNAs exert their biological functions by regulating the expressions of their target genes. Our method was based on this conclusion and we intended to interpret the cause-and-effect mechanism behind the existing miRNA-disease associations based on the extracted component sets. We chose CC31, CC40 and CC54 in the extracted 60 canonical components as examples.

In CC31, we received 3 miRNAs (*hsa-miR-34a*, *hsa-miR-34b* and *hsa-miR-34c*). The numbers of target genes for the 3 miRNAs were 139, 32 and 50, respectively. The top-ranked target gene for the 3 miRNAs was *ZAP70*. Experimental evidence indicated that tyrosine kinase encoded by this gene plays an essential role in regulation, such as immune response, thymocyte development and cytokine expression of mature T-cells. Meanwhile, the top-predicted disease was *sarcoma*, which was a highly malignant tissue neoplasm caused by proliferation of mesodermal cells. Records in the latest version of CTD [39], a publicly available database curated information about environmental factors affecting human health, indicated that *ZAP70* was one of disease genes for *sarcoma*.

Two miRNAs (*hsa-miR-106b* and *hsa-miR-93*) were included in CC40. For *hsa-miR-106b*, it targeted 47 genes and 34 genes were targeted by *hsa-miR-93*. The top predicted genes were *DAB2* and *PTENP1* and the top ranked diseases were *Lung Diseases* and *Ovary Syndrome*. In CTD, we discovered that both genes were related to *Lung Diseases*. Even though, no information was available in CTD about the roles of the two genes in *Ovary Syndrome*. Studies showed that *DAB2* was expressed in normal ovarian epithelial cells and the down-regulation of *DAB2* may lead to ovarian carcinogenesis [40].

In CC54, 5 genes (*ANAPC1*, *RPIA*, *IGF2BP2*, *CYP2J2* and *LRIG1*) and 1 disease (*Cataract*) were extracted as a correlated set. Cataracts often affect old people, causing blurry vision. It was reported that more than half of Americans either have a cataract or have had cataract surgery by the age of 80 (https://www.nei.nih.gov/health/cataract/). Therefore, detecting the genetic causes of cataracts is of great importance. The retrieval of information from CTD suggested that all the 5 target genes were genetic factors of the disease *Cataract*.

It should be pointed out that some records of disease genes in CTD might be inferred. Even though, the co-occurrence of disease genes extracted by our method and recorded in the CTD database reinforced the reality of such information because our knowledge of disease genes was not complete.

### Performance evaluation

To evaluate the prediction ability of our approach, we conducted 5-fold cross validations on the benchmark datasets. We first randomly split the 404 miRNAs into five subsets of roughly equal size. Each subset is then used in turn as a test set and training is performed on the remaining four subsets. For the test set, the related disease relationships of miRNAs are removed and we calculate the prediction scores based on the weight vectors of the components extracted from the training set. We rank the predicted miRNA-disease associations according to the prediction scores. The higher a validated miRNA-disease association is ranked, the better the prediction performance is. To obtain robust results, we repeated the cross-validation experiments five times.

Given a threshold *δ*, if the score of a predicted result is greater than *δ*, it is considered as a positive sample. Otherwise, it is deemed as a negative sample. To obtain a receiver operating characteristic (ROC) curve, the true positive rates (TPRs) and the false positive rates (FPRs) at various *δ* values are computed as,

1 $$ TPR=\frac{TP}{TP+ FN}, FPR=\frac{FP}{TN+ FP} $$

where TP and TN are the numbers of correctly identified positive and negative samples. FP and FN are the numbers of misidentified positive and negative samples. The area under the ROC curve (AUC) is used to measure the performance of our approach.

There are three parameters in our method. The parameters *c*_*1*_ and *c*_*2*_ are to control the sparseness, and *K* is the number of pairs of extracted canonical components. They are well tuned and we obtain the best AUC value of 0.84606 with *c*_*1*_ = 0.01, *c*_*2*_ = 0.01 and *K* = 60.

### Prediction of novel miRNA-disease associations

After confirming the prediction performance by cross validations, we further applied our method to the miRNAs, which was not included in the benchmark datasets but whose target gene information was available, for their disease association predictions. There were 100 miRNAs of such kind in miRTarBase. We considered them as new miRNAs and their associated diseases needed to be predicted. The benchmark datasets were taken as a training set. The predicted results for each new miRNA were listed in Additional file 2.

As we were preparing for the manuscript, the database HMDD v3.0 [41] was released. We took HMDD v3.0 as evidence to confirm the newly predicted miRNA-disease associations. We selected *hsa-miR-203a* as an example. For this miRNA, we chose the top 10 predicted results from the 362 candidate diseases and discovered that 5 of them were annotated by HMDD v3.0 (Table 1).

**Table 1:** The top 10 predicted results for *hsa-miR-203a*

miRNA	rank	predicted disease	confirmed
*hsa-miR-203a*	1	Colorectal Neoplasms	
*hsa-miR-203a*	2	Breast Neoplasms	Yes
*hsa-miR-203a*	3	Stomach Neoplasms	Yes
*hsa-miR-203a*	4	Melanoma	
*hsa-miR-203a*	5	Carcinoma, Hepatocellular	Yes
*hsa-miR-203a*	6	Lung Neoplasms	Yes
*hsa-miR-203a*	7	Prostate Neoplasms	Yes
*hsa-miR-203a*	8	Heart Failure	
*hsa-miR-203a*	9	Carcinoma, Non-Small-Cell Lung	
*hsa-miR-203a*	10	Urinary Bladder Neoplasms	

**Fig. 1:**
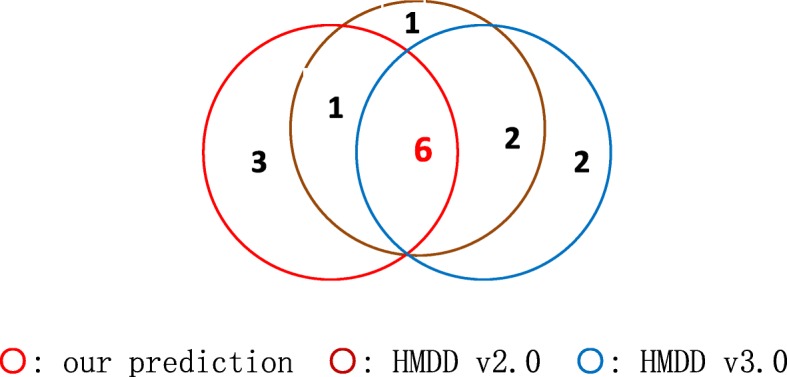
Comparison of the top 10 diseases inferred by our method and the top 10 diseases with the highest values of MSW in HMDD v2.0 and HMDD v3.0

Furthermore, we chose the top 10 diseases (Table 2) predicted by our method and compared them with the top 10 diseases with the highest values of MSW (miRNA spectrum width) [41], an indicator of complexity of miRNA regulation, based on HMDD v2.0 and HMDD v3.0 (Fig. 1). As there are still associations between miRNAs and diseases undetected, the co-occurrence of top diseases in Fig. 1 demonstrates the inference capacity of our method from another way of perspective.

**Table 2:** The top 10 predicted diseases by our method

Rank	Our prediction
1	Carcinoma, Hepatocellular
2	Breast Neoplasms
3	Colorectal Neoplasms
4	Stomach Neoplasms
5	Lung Neoplasms
6	Melanoma
7	Urinary Bladder Neoplasms
8	Ovarian Neoplasms
9	Glioblastoma
10	Glioma

## Discussion

Revealing the roles of miRNAs in diseases is critical for understanding the genetic causes of human diseases. Satisfied prediction performance could be obtained by previous methods for inferring miRNA-disease associations. However, they seldom investigated the underlying cause-and-effect mechanism involved. The correlated sets of target genes and human complex diseases received by our CCA-based method provided insights into the functional roles of miRNAs in the development of diseases. Three case studies also demonstrated correlation between the extracted target genes and diseases.

The excellent performance of our method could be contributed to two factors: data quality and additional penalty imposed on the CCA model. The benchmark datasets were received from two highly reliable databases and the miRNA-gene interactions and miRNA-disease associations were supported by published papers. Furthermore, adding more penalties on ordinary CCA produced sparse weight vectors, which facilitate reasonable interpretation of the results.

As the data features used in our method were different from those in other computational models, a direct performance comparison was hard to implement. It is supposed in our method that a new miRNA’s target gene information is available, but not disease information. Indeed, it is not practical in a real situation that the detailed target gens profile is known for a miRNA candidate molecule, which is a disadvantage in our method. However, advance in biological assays is bringing increasing information regarding miRNA-gene interactions. In this context, we expect that our proposed method presents itself as an informative tool for discovering the pathogenesis of diseases.

## Conclusions

In this paper, we developed a novel method based on CCA with sparseness constraints for inferring and interpreting miRNA-disease associations. The results received from cross validations confirmed the excellent prediction power of our method. Experimental results also indicated that imposing the sparseness characteristic on CCA contributed to the improvement of interpretation of miRNA-disease associations. The extracted pairs of genes and diseases offered biological guidance for investigating how miRNA-disease associations were formed. When applying our method for predicting associated diseases for new miRNAs, some high-scoring results were supported by HMDD v3.0.

## Methods

### Data preparation

Two datasets, namely miRNA-gene interactions and miRNA-disease associations, were used in our method. We downloaded miRNA-gene interactions from miRTarBase [42], which was built by manually surveying pertinent literature to retrieve experimentally confirmed miRNA-gene interactions. For these interactions, we constrained the miRNA species into *Homo sapiens*. The interactions supported by weak experimental evidences were not taken into consideration. miRNA-disease associations were received from HMDD v2.0 [14] whose experimentally validated associations between miRNAs and diseases were manually retrieved from literature. For both datasets, we merged the records of different miRNA copies that produce the same mature miRNA. Diseases with synonyms in HMDD v2.0 were also merged and invalid disease names were filtered out. Redundant records in the two datasets were kept only once.

**Fig. 2:**
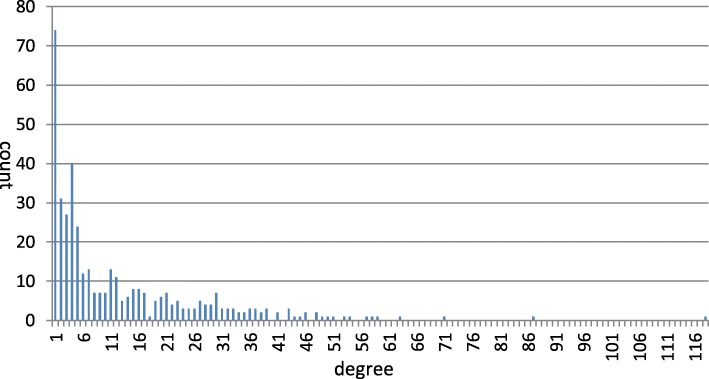
The degree distributions of miRNAs in miRNA-disease association dataset

**Fig. 3:**
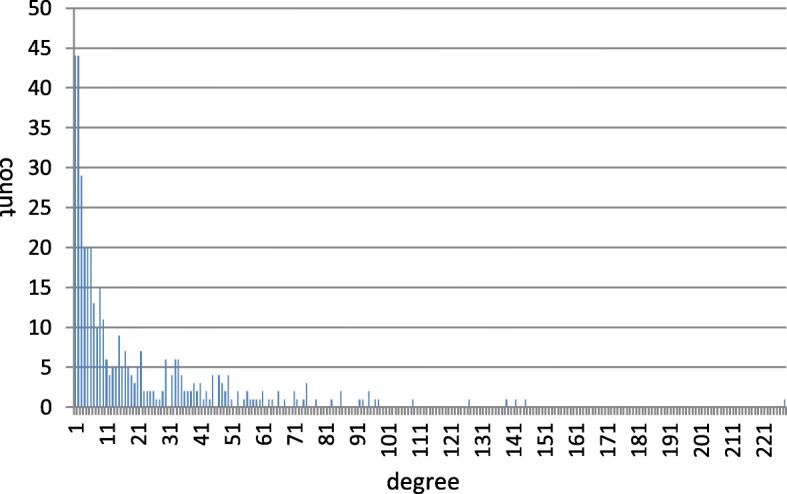
The degree distributions of miRNAs in miRNA-gene interaction dataset

In total, 404 miRNAs had both target gene and disease information. We finally received 7999 miRNA-gene interactions containing 2796 target genes and 5117 miRNA-disease pairs containing 362 diseases (Table 3). Intuitively, we could model each of the two datasets as a bipartite graph. The degree distributions of miRNAs, which indicate the numbers of target genes or the numbers of associated diseases of miRNAs, in each graph were listed in Fig. 2 and Fig. 3, respectively. The two sets of data were regarded as benchmark datasets and were used for performance evaluation in the following cross-validation experiments as well as training data for novel miRNA-disease association predictions.

**Table 3:** Statistics of the datasets used in our manuscript

Name	Statistics
# miRNAs	404
# target genes	2796
# diseases	362
# miRNA-gene interactions	7999
# miRNA-disease associations	5117
average number of target genes for each miRNA	19.8
average number of related diseases for each miRNA	12.7

### Method description

#### Construction of gene profiles and disease profiles for miRNAs

Suppose that we have a set of *n* miRNAs which included *p* target genes and *q* related disease features. Each miRNA can then be denoted by a target gene profile t = (t_1_, t_2_, t_3_, …, t_*p*_)^*T*^ and by a disease profile *d* = (d_1_, d_2_, d_3_, …, d_*q*_)^*T*^, where t_*i*_ (or d_*j*_) is represented for the presence or absence of gene (or disease) by 1 or 0, respectively.

#### Canonical correlation analysis (CCA)

Two linear combinations for target gene profiles and disease profiles are defined as *u*_*i*_ = *α*^*T*^*t*_*i*_ and *v*_*i*_ = *β*^*T*^*d*_*i*_ (*i* = 1,2,3,…,*n*), where *α* = (*α*_1_, *α*_2_, *α*_3_, …, *α*_*p*_)^*T*^ and *β* = (*β*_1_, *β*_2_, *β*_3_, …, *β*_*q*_)^*T*^ are weight vectors. Our goal is to find weight vectors *α* and *β* which maximize the following canonical correlation coefficient:

2 $$ \rho = corr\left(u,v\right)=\frac{\sum_{i=1}^n{\alpha}^T{t}_i\bullet {\beta}^T{d}_i}{\sqrt{\sum_{i=1}^n{\left({\alpha}^T{t}_i\right)}^2}\sqrt{\sum_{i=1}^n{\left({\beta}^T{d}_i\right)}^2}} $$

where *u* and *v* are centered, respectively.

Let *X* denote the *n × p* matrix and *Y* denote the *n × q* matrix. Then the maximization problem can be formally rewritten as follows:

3 $$ \max imize\left\{{\alpha}^T{X}^T Y\beta \right\}\;\mathrm{subject}\ \mathrm{to}\ {\left\Vert \alpha \right\Vert}_2^2\le 1,{\left\Vert \beta \right\Vert}_2^2\le 1. $$

Canonical correlation analysis (CCA), developed by Hotelling [43], provided a solution to the problem. We consider it as ordinary canonical correlation analysis (OCCA). However, OCCA usually results in vectors *α* and *β* that are not sparse. We are interested in finding a linear combination of the variables in *X* and *Y* that has large correlation but is also sparse in the variables used. We therefore choose to add penalties in (2) and reconsider the maximization problem as:

4 $$ \max imize\left\{{\alpha}^T{X}^T Y\beta \right\}\;\mathrm{subject}\ \mathrm{to}\;{\left\Vert \alpha \right\Vert}_2^2\le 1,{\left\Vert \beta \right\Vert}_2^2\le 1,{\left\Vert \alpha \right\Vert}_1\le {c}_1\sqrt{p},{\left\Vert \beta \right\Vert}_1\le {c}_2\sqrt{q} $$

where *c*_*1*_ and *c*_*2*_ are parameters to control the sparsity. We refer to this as sparse canonical correlation analysis (SCCA).We applied a strategy of penalized matrix decomposition (PMD) [44] on the matrix *Z* = *X*^*T*^*Y* to obtain the weight vectors *α* and *β*.

To compute multiple canonical variates, we performed PMD on the following *Z*^*k*^ iteratively.

1. Let *Z*^1^ = *X*^*T*^*Y*
2. For *k* ∈ 1, 2, …, *K* − 1

${Z}^{k+1}={Z}^k-{d}_k{\alpha}_k{\beta}_k^T$ (*α*_*k*_ and *β*_*k*_ are the weight vectors, and *d*_*k*_ is singular value obtained in each step).

Similar to [45], we selected genes and diseases in the *K* pairs of weight vectors with the highest values as correlated sets.

#### Novel miRNA-disease association predictions

To predict the related disease profile *y*_*new*_ of a new miRNA with a known target gene profile *x*_*new*_, we calculate the prediction scores based on the *K* pairs of weight vectors received above according to the following equation:

5 $$ {y}_{new}=\sum \limits_{k=1}^K{\beta}_k{\rho}_k{\alpha}_k^T{x}_{new} $$

**Fig. 4:**
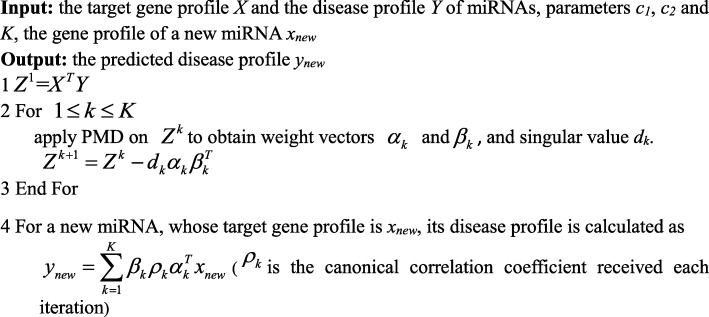
Description of the algorithm SCCA

**Fig. 5:**
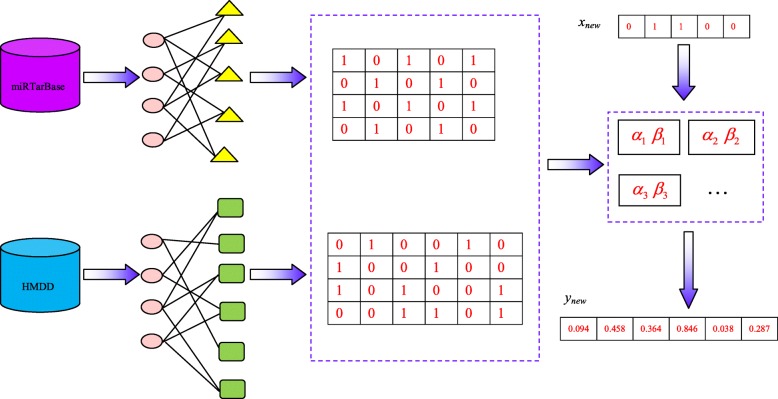
Schematic of our proposed model. First, we extracted miRNA-gene interactions and miRNA-disease associations from miRTarBase and HMDD, respectively. Then, target gene profiles and disease profiles for miRNAs were constructed. Third, canonical correlation analysis was performed to obtain correlated sets. Finally, novel miRNA-disease associations were predicted based on the weight vectors of the correlated sets.

The elements in *y*_*new*_ with the highest scores are chosen as the potential diseases for the new miRNA. We outlined the complete steps for inferring potential miRNA–disease associations in Fig. 4, and the workflow of our model was illustrated in Fig. 5.

## Additional files

Additional file 1 The extracted 60 canonical components. (TXT 25 kb)

Additional file 2 The predicted scores of diseases for the 100 new miRNAs. (XLS 427 kb)

## Data Availability

The datasets used and/or analysed during the current study are available from the corresponding author on reasonable request.

## Abbreviations

CC: Canonical component
CCA: Canonical correlation analysis
miRNAs: microRNAs
MSW: MiRNA spectrum width
PMD: Penalized matrix decomposition
